# Testing the Decision Support Tool for Responsible Pain Management for Headache and Facial Pain Diagnosis with Opioid-Risk-Stratified Treatment

**DOI:** 10.1007/s42399-023-01423-1

**Published:** 2023-02-27

**Authors:** Barbara St. Marie, Yelena Perkhounkova, Amalia Gedney-Lose, Andrea Jimmerson, Brooke Porter, Keela Herr, Prakash Nadkarni

**Affiliations:** 1 College of Nursing, University of Iowa, 50 Newton Road, Iowa City, IA 52242 USA; 2 College of Nursing, Biostatistics, University of Iowa, 50 Newton Road, Iowa City, IA 52242 USA; 3 Internal Medicine, University of Iowa, Iowa City, IA 52242 USA

**Keywords:** Headache, Facial pain, Clinical decision support, Vignettes, Opioid use disorder

## Abstract

In primary and urgent care, headache and facial pain are common and challenging to diagnose and manage, especially with using opioids appropriately. We therefore developed the Decision Support Tool for Responsible Pain Management (DS-RPM) to assist healthcare providers in diagnosis (including multiple simultaneous diagnoses), workup (including triage), and opioid-risk-informed treatment. A primary goal was to supply sufficient explanations of DS-RPM’s functions allowing critique. We describe the process of iteratively designing DS-RPM adding clinical content and testing/defect discovery. We tested DS-RPM remotely with 21 clinician-participants using three vignettes—cluster headache, migraine, and temporal arteritis—after first training to use DS-RPM with a trigeminal-neuralgia vignette. Their evaluation was both quantitative (usability/acceptability) and qualitative using semi-structured interviews. The quantitative evaluation used 12 Likert-type questions on a 1–5 scale, where 5 represented the highest rating. The mean ratings ranged from 4.48 to 4.95 (SDs ranging 0.22–1.03). Participants initially found structured data entry intimidating but adapted and appreciated its comprehensiveness and speed of data capture. They perceived DS-RPM as useful for teaching and clinical practice, making several enhancement suggestions. The DS-RPM was designed, created, and tested to facilitate best practice in management of patients with headaches and facial pain. Testing the DS-RPM with vignettes showed strong functionality and high usability/acceptability ratings from healthcare providers. Risk stratifying for opioid use disorder to develop a treatment plan for headache and facial pain is possible using vignettes. During testing, we considered the need to adapt usability/acceptability evaluation tools for clinical decision support, and future directions.

## Background

The USA continues to face a burden of opioid misuse and pain management including facial pain and headache. Over 2.1 million people aged 12 or older have opioid use disorder (OUD) [1]. A 40% increase in overdose deaths (*n* = 81,000) occurred between mid-2019 to mid-2020 [2]. Prescription opioids, involved in over 28% of all opioid overdose deaths in 2019, contribute to the healthcare crisis [3–5].

Severe headaches, affecting 16% of the US population [6] and up to 70% of patients with COVID-19 [7, 8], result in 4.3 million ambulatory-care visits yearly [6] and are the fifth most common reason for emergency department (ED) visits [9]. Opioids are currently used in over 50% of migraine visits in the ED [10, 11]. ED patients receiving opioids have higher risk for recurrent opioid use 1 year later [12–15]; however, predicting opioid misuse is challenging.

Headache misdiagnosis is common, notably for migraine (often misdiagnosed as “sinus headache” [16, 17] and cluster headache [18–20]. The Chronic Migraine Epidemiology and Outcomes (CaMEO) Study found that only 5% of migraine patients receive both correct diagnosis and guideline-recommended treatment [21]. In a large transcontinental cohort (*n* = 1161), 50% of patients were imaged unnecessarily [22]. Diagnostic errors can result in mistreatment, long-term co-morbidities [23], and empirical pain-suppression approaches rather than superior disease-specific therapy.

Many healthcare providers (HCP) lack knowledge of current pain management guidelines or legal liability when managing pain [24–26]. Existing opioid-use guidelines [27] and pain-management guidelines [28, 29] or algorithms [30] have the following actual or perceived limitations:

- *Excessively siloed:* Pain-management content is not integrated with opioid misuse prevention recommendations [31–33], making it challenging to address chronic/severe pain management with possible concurrent opioid misuse.
- *Too voluminous/complex* to recall easily for routine clinical care.
- *Not updated frequently* with changing knowledge/regulatory environments.

American primary care HCPs face extreme time pressure and cognitive burden, with approximately 2 min to address each major health concern, with even less time for minor ones [34]. Furthermore, the vast medical knowledge relevant to primary care evolves continually. In complex, hurried circumstances, decision-making matches what Herbert Simon called “Bounded Rationality” [35]: decision-makers tend to take mental short-cuts (“heuristics”) to attain “satisfactory” rather than optimal outcomes. Behavioral economists Daniel Kahneman and Amos Tversky showed that short-cut thinking is bias and error prone [36]. Therefore, advice on using opioids safely must be coupled with assistance in accurate diagnosis, to determine whether opioids are indicated—as a last resort, or at all. Our purposed software solution is to create the Decision Support Tool for Responsible Pain Management (DS-RPM) to reduce or prevent opioid misuse, reduce misdiagnosis and treatment errors, and ease cognitive burden of HCPs.

### Purpose

The purpose of this study is to explore delivery of an electronic clinical decision support (i.e., DS-RPM) to HCPs for facial pain and headache diagnosis, workup plus opioid-risk management. At the point of care, DS-RPM is designed to diagnose facial pain and headache and assists the HCP to develop a risk-stratified treatment plan with potential to reduce opioid misuse. There are diagnostic challenges that the DS-RPM is potentially capable of resolving: (a) *identifying multiple diagnoses* when present in the same patient, (b) provide differential diagnoses in unclear cases, and (c) determine differential diagnostic workup recommendations such as a “therapeutic trial” of indomethacin for paroxysmal hemicrania and hemicrania continua. The DS-RPM can also assist with *“intermediate diagnoses”* with varied root causes. For example, optic neuritis and trigeminal neuralgia may respectively be the presenting symptom of multiple sclerosis (MS) in 15–20% [37] and 15% [38] of MS patients. Similarly, cranial neuralgias and cluster headache may be caused by neoplasia and vascular abnormalities [39]. DS-RPM can *recommend immediate triage or referral to emergency or specialty care* when specific “red-flag” findings on history or examination suggest conditions too critical for primary-care management. *Explanations are provided regarding DS-RPM’s diagnostic process* and include online references relevant to suggested diagnoses, prioritizing references with freely accessible full text. These explanations are necessary for HCPs to trust DS-RPM, as well as allow HCPs to provide feedback by informatively disputing the process for specific diagnoses. The DS-RPM enables *selection for a customized treatment plan.* Following diagnosis, interventions are ordered by preference in the management-guideline literature.

DS-RPM is considered an advanced *functional prototype* in that it has most features expected of a production application. However, there are some deliberate limitations:

- *Restricted scope*: We limited ourselves to headache and facial pain rather than all pain locales because it is both common and challenging in outpatient and primary care. Addressing it even in a prototype provides value to HCPs and can incentivize them to provide detailed, sustained feedback.
- *Inability to fix problems for numerous concurrent users or to interoperate fully with the electronic health record (EHR)*: We deliberately chose to focus on scientific issues rather than extensive software engineering efforts, which are inappropriate for a prototype. However, to reduce duplicative data entry in the EHR, DS-RPM generates semi-structured narrative text which can be copied and pasted into the EHR’s note fields. This narrative text summarizes positive and significant negative findings, suggested diagnosis or differential, and recommendations for further workup and/or treatment.

## Methods

### Overview

An explanation of DS-RPM development and commenting on the features is necessary as it sets the groundwork for this study. Development included establishing knowledge content, choice of rapid-prototyping technology, the user interface, create computable content, and use vignettes for iterative testing. Our institutional review board approved DS-RPM’s development and its evaluation by human subjects (Midwest primary-care HCPs), whose recruitment involved informed consent. The study was considered minimum hazard, no patients were involved, and criticism of DS-RPM was encouraged, and thereby granted exemption.

#### Development of DS-RPM Knowledge Content

DS-RPM currently handles the conditions listed in Table 1. Its algorithms were assembled from numerous online sources such as diagnostic algorithms (including differential diagnosis) and therapeutic interventions. We prioritized review articles, expert-panel papers, meta-analyses, selected clinical trials, plus case reports for uncommon conditions or atypical presentations. Our bibliography currently has over 300 references, categorized by reference type and areas of focus: diagnosis, differential diagnosis investigations, treatment, and patient education. We prioritize non-paywalled online content (e.g., PubMed + PubMed Central, Medscape) that is readily workplace accessible.

**Table 1:** Primary diagnoses currently handled in DS-RPM

Tension-type headache
Migraine and its various presentations (e.g., with/without aura)
Cerebrovascular accident (CVA) and its subcategories
Intracranial space-occupying lesions of various etiologies (neoplasia, vascular, acute/chronic pituitary disorders)
Trigeminal autonomic cephalalgias—cluster headache, paroxysmal hemicrania, hemicrania continua, short-lasting unilateral neuralgiform headache with autonomic symptoms/conjunctival tearing (SUNA/SUNCT)
Neuralgic disorders: trigeminal neuralgia, glossopharyngeal neuralgia, occipital neuralgia, post-herpetic neuralgia
Systemic causes of headache: e.g., temporal (giant-cell) arteritis, pheochromocytoma, lupus erythematosus
Disorders of the salivary glands—sialadenitis, sialadenosis, neoplasia, Sjogren–Mikulicz disease
Temporomandibular joint disorders
Disorders of the paranasal sinuses
Infective causes of CNS inflammation—meningitis, encephalitis, HIV, Lyme disease
Toothache and its differential/root causes: Caries, abscess, trigeminal neuralgia, etc
Optic neuritis and its root causes (e.g., multiple sclerosis, neuromyelitis optica)
Ophthalmic causes of headache/facial pain (e.g., uveitis, keratoconjunctivitis, refractive errors)
Cavernous sinus disorders—thrombosis, Tolosa–Hunt syndrome

Our starting point for headache/facial pain was ICHD-3 (International Classification of Headache Disorders, 3rd Edition) [40]. While useful, we discovered numerous issues limiting its utility which is presented in the discussion section. We used several well-known questionnaires to quantify risk scores, which were used as the basis for customized recommendations:

- *Opioid risk* (with the creators’ permission): the 8-item Revised (2019) Opioid Risk Tool for Opioid Use Disorder (ORT-OUD) [41]. The ORT-OUD Risk Score is used to generate a risk-stratified treatment plan for opioid use where appropriate.
- *Depression*: Chronic pain can cause depression. The 9-item Patient Health Questionnaire (PHQ) is activated if the patient responds to either of two questions in the history (“Little pleasure in doing things”; “Feeling down, depressed, or hopeless”) with “More than half the days” or worse [42].
- *Suicide risk:* The National Institute of Mental Health’s 4-item ASQ (Ask Suicide Screening Questions) scale is activated after a non-negative response to the PHQ question “Thoughts that you would be better off dead, or of hurting yourself.” Chronic pain is an important risk factor for suicide [43]: chronic headache posed higher suicide risk than other chronic pain types in a 10-year veterans’ retrospective study [44].

#### Choice of Rapid-Prototyping Technology

We used Microsoft Access which includes a Structured Query Language (SQL)–capable Relational Database engine plus a built-in interpreted programming language, Visual Basic for Applications, which is used across all Microsoft Office applications. Access supports iterative cycles of rapid application development: the graphical user interface (GUI) is created visually by dragging/dropping objects from a palette and adding code snippets to individual objects as needed. An Access database is deliberately limited (by Microsoft) to 2 GB, but is readily upsized, after the prototype reaches stability, to a high-end database (e.g., Microsoft SQL Server, MySQL) with the application now acting as a front-end. This hybrid setup supports about 30 concurrent users within an Intranet. For wider-scale deployment, the GUI must be migrated to a Web platform [45].

#### The User Interface

**Fig. 1:**
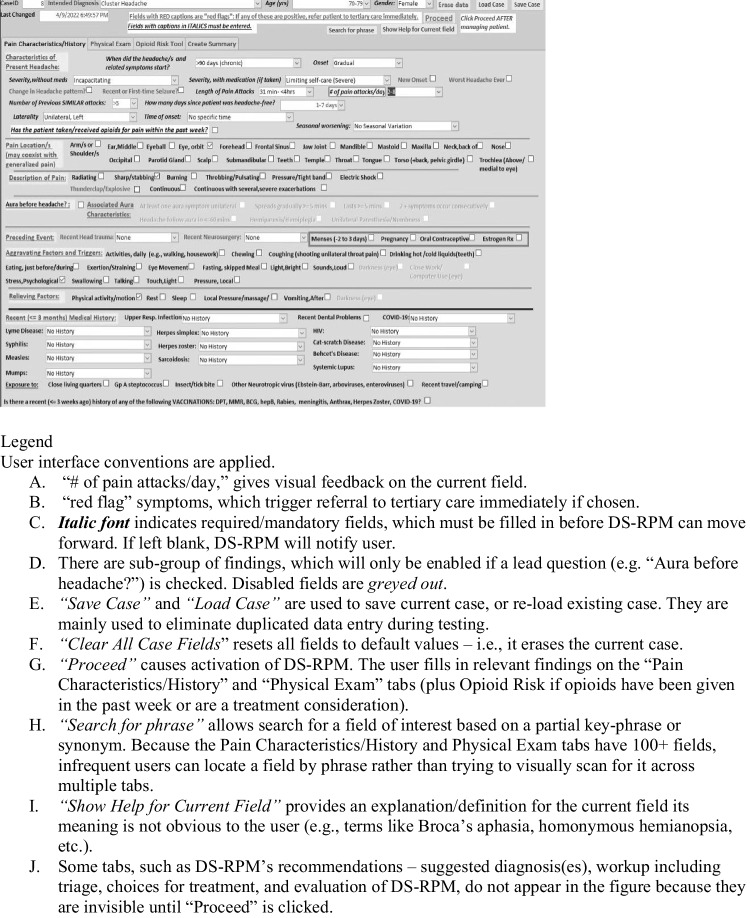
Screenshot of characteristics/history tab of DS-RPM

Figure 1 shows a screenshot (see legend for Fig. 1). The interface is organized into “tabs” for the following purposes:

- *Entering pain characteristics/history and physical examination findings.* Input uses *structured data entry* for individual symptoms/signs presented mostly as checkboxes (for Yes/No items) or pull-down menus with reasonable defaults. Only a few findings, e.g., systolic/diastolic BP and temperature, are entered numerically. Validation of input and entry speed is facilitated through “skip logic” enabling or disabling certain items based on responses to certain preceding question. For example, the aggravating factors “Menstrual period” and “Oral Contraceptives” are disabled for a male patient. To assist user navigation of the interface, DS-RPM supports search for specific questions by (partial) keyword, including synonyms.
- *Displaying recommendations* of diagnosis(es) and differential, and presenting users with therapeutic options for the final diagnosis.
- *Calculating risk scores.* These are risk scores for screening depression, suicide, and opioid use disorder. Risk scores tools appear on the Physical Exam tab and Opioid Risk Tool tab.
- Allowing the user to *save the current case’s findings, recommendations, and therapy selections*. These are used to generate summary text output. The summary text can be copied and pasted into EHR.
- Letting the user *provide evaluator feedback* on DS-RPM by clicking on *Evaluate Case.*

DS-RPM operates in two user-interface modes: developer/author mode and end-user mode. Developer or author mode allows the development team members to add or edit knowledge content. End-user mode occurs when the user enters a set of findings for which a recommendation is desired. End-users are prevented from modifying knowledge content and many developer mode actions are disabled or invisible.

#### Create Computable Content

The knowledge content is operationalized using branching logic (yes/no or multi-way choices) augmented by a software library that allows the logic to be expressed compactly. For example, risk-score calculation uses a library function that simply sums up the values of an arbitrary list of items. Most of the system’s knowledge is represented in tables rather than program code. Database technology allows efficient location of items of interest using B-tree indexing [46]. We also incorporate *alerting mechanisms* to prompt HCP actions. Example alerts include.

- Triage recommendation, which typically suggests the diagnosis(es) being considered.
- Prompt to complete the ORT-OUD for patients who have received opioids during the last week or are expected to receive opioids for their pain.
- Recommend immediate supervised tertiary-care transfer for gravely ill or suicidal patients.

DS-RPM includes a rule engine [47] and, with some programming effort, an open-source engine like JBoss Drools [48] could substitute.

#### The Use of Vignettes

Widely used for teaching, a *vignette* is a set of findings describing a hypothetical patient with a particular diagnosis/differential. Well before DS-RPM’s design, we created seven prose vignettes for pain etiologies and evaluated their content validity [49]. Users evaluate DS-RPM’s diagnostic accuracy and usability by entering findings from validated vignettes for specific diagnoses.

*DS-RPM represents vignettes electronically* as a set of clinical-variable values identified by an intended diagnosis(es) or differential, plus a text description. These are all entered by a development-team member and saved. We reuse saved vignettes for user training and regression testing to eliminate having to re-enter findings manually.

### Study Design

#### Recruitment

Participants were recruited for the two phases of beta testing, early beta and late beta. Inclusion criteria for participants involved for each beta tests included (1) English speaking, (2) currently in clinical practice with prescriptive authority and DEA licensure, (3) available for 1–2 hours using Zoom, a videoconferencing platform, with the DS-RPM and the PI on a shared screen, and (3) willing to participate in an audio-recorded interview by phone for 30 minutes. Recruitment occurred through the American Society for Pain Management Nursing (early beta) and University of Iowa primary practice clinics and faculty practice (late beta). During late beta recruitment, 43 qualifying clinical practice providers were contacted through email; however, 49% contacted the PI and agreed to participate.

#### Testing and Evaluation of DS-RPM

Testing identifies defects which can be fixed. We followed standard methodology in testing DS-RPM at the level of both individual components (e.g., *unit tests*, which exercise individual functions within the code) and the entire system *(integration testing*). The latter is typically divided into two phases, alpha testing and beta testing [50]. Alpha testing is performed internally, occurred in the early phases of development within the development team. The development team tested numerous scenarios based on clinical experiences and changes were made based on performance of DS-RPM. Beta testing is performed external to the development team, using vignettes and occurred in two steps: early beta and late beta. In *early beta testing*, a few highly committed external testers (*n* = 3) explored the software in-depth with vignettes and provided detailed feedback. Three recruited participants were all female with 9 to 25 years pain management experience. Two participants had Masters’ degree and one had a PhD in Nursing. In *late beta testing*, multidisciplinary clinicians (*n* = 21) tested under more diverse conditions. The late beta testing diverged from traditional software testing because participants not only identified defects, but also evaluated DS-RPM quantitatively and qualitatively. This evaluation design was informed by lessons learned during alpha and early-beta testing. Thus, the results section reports only on the late-beta phase.

*Regression Testing.* Software modifications to fix defects or add new features may themselves introduce new defects. *Regression testing* [51] is employed throughout software development, minimizes risk for introducing new defects, uses a suite of tests that exercises all aspects of the software, and is ideally executed automatically/semiautomatically after a modification. Every test’s actual output is compared with the expected output. Discrepancies help identify and localize defects. As the software evolves, new tests are added. Complex software uses hundreds to thousands of tests.

DS-RPM additionally uses electronic vignettes for regression testing. For each electronic vignette, the expected outputs include the intended diagnosis(es) and differential, the suggested workup and interventions, and the suggested bibliography; and if opioids were used, suggestions for stratifying risk, and subsequent treatment for OUD. We update knowledge content regularly; for example, calcitonin-gene-related-peptide inhibitors for migraine were introduced after this project commenced. Therefore, the expected outputs change.

*Defect Discovery.* There were four categories of defects discovered and were fixed over the course of alpha and beta testing. *Logic defects* created software crashes and erroneous output. *Ergonomic defects* transpired when common tasks require too many steps, questions were placed in seemingly random order, and help was needed for remembering one’s place if data entry was interrupted. These were immediately fixed by creating shortcuts, rearranging questions into logical groups, and highlighting the current work field in cyan, respectively. *Content-related defects* resulted in unclear captions for fields, confusing or inaccurate explanations, redundancies or duplicates among clinical variables, and references not deemed useful. *Cosmetic defects* were spelling errors, confusing layout, fields misaligned, and excess spacing between labels and corresponding data-entry fields. While we fixed defects expeditiously, software updates incorporating fixes to non-logic defects were batched to avoid frequent user-interface changes, which can confuse users.

### Data Collection and Analysis

All participants were trained individually at their convenience by the first author with remote-control-enabled Zoom using a trigeminal neuralgia vignette. They later entered data and executed DS-RPM for each test vignette by themselves under the first author’s observation. After each vignette, they evaluated DS-RPM on functional requirement criteria. After finishing all vignettes, they completed the usability and acceptability evaluation. A 30-min audio-recorded interview followed to inform us how DS-RPM could be improved. Interview guide is available upon request.

DS-RPM’s quantitative evaluation criteria (Table 2) fall into three categories:

- *Functional Requirements* relate to DS-RPM’s intended purpose, i.e., triage, suggested diagnosis, treatment, opioid risk, explanations, references, and error messages. Because DS-RPM’s coverage depth for individual diagnoses may vary, these criteria were evaluated separately for each of three test vignettes: temporal (giant-cell) arteritis, migraine, and cluster headache.
- *Usability* is the extent to which DS-RPM is understandable, learnable, efficient to operate, and free from spelling and grammar errors.
- *Acceptability* is the extent to which DS-RPM is perceived to meet its intended purpose, and whether a user would use it regularly and recommend it to others.

**Table 2:** Evaluation criteria for DS-RPM^1^

Criterion	Item
Functional requirements	Diagnosis(es) suggested for the given case were appropriate
	Treatments suggested for the diagnosis were appropriate and evidence based
	Explanations for triage, diagnosis, and treatment were useful and appropriate
	References suggested were appropriate and current
	Error messages were useful and appropriate
Usability	The decision support tool was understandable
	The tool was easy to learn to use
	The tool was efficient to operate when used repeatedly
	The interface and output were grammatically correct without spelling errors
Acceptability	Overall, the tool functions as intended
	I would use this application in my practice
	I would recommend this application to my colleagues

^1^Questions use a Likert Scale: 1 = disagree strongly; 2 = disagree somewhat; 3 = neutral; 4 = agree somewhat; 5 = agree strongly, 0 = not applicable. Each item is accompanied by a text field of unlimited size where the user can elaborate on their preceding response

We based usability and acceptability criteria on the well-known System Usability Scale (SUS) [52] but adapted or abbreviated for reasons explained in the discussion. All criteria were graded on a 1–5 Likert scale: 1 = disagree strongly, 2 = disagree somewhat, 3 = neutral, 4 = agree somewhat, 5 = agree strongly, and 0 = did not apply to case.

The criteria were presented to the user on an electronic form in the “Evaluate Case” tab of the GUI, and data was analyzed using mean and standard deviation of each category. Each Likert-scale question was accompanied by a narrative-text “Comment” field where users could clarify reasons for their response, identify problems, and suggest improvement.

Qualitative data analyses of comment fields in the evaluation form and audio interviews were inductive and informed by qualitative procedures [53–56]. Audio transcripts were read and coded by two members of the research team, who met weekly to arrive at consensus from open coding to sub-categories to categories to themes.

## Results: Late Beta Testing

### Quantitative Data

Late beta testing occurred from July to September 2021. Table 3 summarizes the clinicians’ demographics. Functional requirement ratings using three vignettes (cluster headache, acute migraine, temporal [giant-cell] arteritis) were all close to the maximum of five and are displayed in Table 4. DS-RPM’s usability and acceptability ratings are shown in Table 5 (A and B, respectively). Mean ratings ranged from 4.76 to 4.95 (*SDs* ranging 0.22–0.62) for usability and between 4.48 and 4.95 (*SDs* ranging 0.22–1.03) for acceptability, indicating high usability and acceptability of DS-RPM. On the criterion, “I would use this application in my practice,” the lower mean score of 4.48 (*SD* ± 1.03) may relate to location or specialty of participant practices. Six of the 21 participants were experienced tertiary-care pain specialists.

**Table 3:** Participant characteristics: late beta testing

Variable	Mean ± SD (Range)	
Years in practice	13.6 ± 11.8 (1.0–41.0)	
	*n*^a^	%
Education		
Doctor of Nursing Practice	16	76.2^b^
Doctor of Medicine	2	9.5^c^
Physician Assistant	2	9.5
Master’s in nursing	1	4.8
Gender		
Female	16	76.2
Male	5	23.8
Race		
White	19	90.5
Asian	2	9.5

^a^*N* = 21

^b^4 in pain-management practice

^c^1 neurologist, 1 anesthesiologist

**Table 4:** Mean measurements of function

	Vignettes		
	Cluster headache	Acute migraine with aura	Temporal (giant-cell) arteritis
	Mean ± SD	Mean ± SD	Mean ± SD
Functional requirements: evaluation criterion			
1. Triage was suggested appropriately	4.90 ± 0.44	4.90 ± 0.30	4.90 ± 0.30
2. Diagnosis(es) suggested for the given case were appropriate	5.00 ± 0.00	4.90 ± 0.30	5.00 ± 0.00
3. The treatments suggested for the diagnosis were appropriate and evidence based	4.76 ± 0.70	4.76 ± 0.70	4.95 ± 0.22
4. Explanations for triage, diagnosis, and treatment were useful and appropriate	4.85 ± 0.49	4.89 ± 0.32	4.94 ± 0.24
5. References suggested were appropriate and current	4.81 ± 0.40	4.70 ± 0.73	4.90 ± 0.30

**Table 5:** Mean measurements of usability (A) and acceptability criterion (B)

	Mean ± SD
A. Usability: evaluation criterion	
1. The decision support tool was understandable	4.81 ± 0.40
2. The tool was easy to learn to use	4.95 ± 0.22
3. The tool was efficient to operate when used repeatedly	4.76 ± 0.62
4. The interface and output were grammatically correct without spelling errors	4.95 ± 0.22
B. Acceptability: evaluation criterion	
1. Overall, the tool functions as intended	4.95 ± 0.22
2. I would use this application in my practice	4.48 ± 1.03
3. I would recommend this application to my colleagues	4.71 ± 0.64

### Qualitative Data

Participants found the recruitment process very easy, and the explanation of the study was thorough. The PI created a recruitment video; however, most participants (81%) did not watch the video because they did not want to spend the time, did not know it was in the recruitment email, or the email information was enough and they already knew they wanted to participate in a study on this topic. Those who did watch it found the video to be helpful in their decision to participate, took a short time to watch, and found it relaxing and personal. Five themes emerged from the analysis: user interface/structured data entry, educational use, training, use of treatment options, and future enhancements suggested by participants.

#### User Interface/Structured Data Entry

Participants felt that the presentation of differential diagnoses, as well alerts for emergency situations, would improve the quality of patient care. Initially, participants found the numerous fields on the “Pain Characteristics/History” and “Physical Exam” tabs busy and intimidating, suggesting numerous user-interface suggestions that we incorporated (see previous explanation of defect discovery). But after testing with two to three vignettes, they found that the organization of questions into logical groups and the use of defaults made data-entry flow comfortable. Participants liked DS-RPM’s structured data entry based on results from the survey and interviews. Likert scale questions that characterize structured data entry are “The tool was efficient to operate when used repeatedly” (4.76, *SD* ± 0.62) and “Overall, the tool functions as intended” (4.95, *SD* ± 0.22). An example of a participant statement is, “If a patient comes into practice, they have a headache, pull up the support tool and just walk through it with the patient as you evaluate.” Another participant liked how the structured data entry cues HCPs to perform sub-examinations that may be missed or forgotten, such as an optic disc examination.

#### Educational Use

Participants saw the potential of DS-RPM to educate HCPs inexperienced in headache management or managing patients with high opioid-misuse risk. Also, DS-RPM’s embedded ORT-OUD screening aligned with their healthcare systems’ opioid-safety initiatives. Nine participants stated that vignettes were helpful, well designed, worked well with DS-RPM, and could be used for web-based interprofessional education.

#### Training

Participants suggested training materials to reduce training burden and improve confidence in using DS-RPM in practice. These materials are recommended in electronic overview and video tutorials*.* An electronic overview can display how DS-RPM provides evidence-based guidelines for diagnoses and treatment options. Video tutorials can show how to navigate the tabs on DS-RPM screens, ask questions from DS-RPM, complete answers based on what patients say, obtain diagnosis and differentials, complete the ORT-OUD, click on the treatment options, and create narrative summaries which can be pasted in the EHR.

#### Use for Treatment Options

The participants appreciated DS-RPM’s targeting of the challenging condition of headaches because there is a lack of access to neurologists, headache specialists, or pain clinics in many healthcare situations. Misdiagnosis may follow a vague patient description of symptoms, resulting in harm and legal liability. Participants thought that DS-RPM use would reassure providers they would cover the possibilities for uncommon or complex headaches.

Participants stated three benefits of using the DS-RPM: (a) DS-RPM directed the history and examination with focus on pertinent positive/negative findings; and assisted in diagnosis, opioid risk stratification, and treatment options; (b) if unsure about a patient, DS-RPM could be accessed faster than looking up symptoms and treatments from other resources; and (c) DS-RPM prioritized a display of treatment options (first choice, second choice, etc.) allowing HCPs to choose alternative treatments, depending on what has been tried. Participant input on the use of the DS-RPM provided the research team with suggestions to improve the decision support tool.

#### Future Enhancements Suggested by Participants

Participants raised the following concerns/suggestions:

- Have more expert review and endorsements of DS-RPM to improve confidence in its use.
- Excessive HCP–computer interaction could adversely affect patient–provider encounter.
- Allow patients to complete some questions as self-assessment before the encounter.
- Expand DS-RPM to thoraco-abdominal pain after DS-RPM’s efficacy in headache/facial pain is established.

## Discussion

Decision-support technology, originally used in non-medical contexts, began evolving in the 1970s. Van Bemmel et al. [57] discussed early work in medical clinical decision support (CDS) methodology, while Giuse et al. [58] addressed medical-knowledge-acquisition approaches. This technology is now mature and mainstream: e.g., Epic uses a rule engine for clinical alerts, which non-programmer informaticians create using a GUI. In developing DS-RPM, we were less concerned with technological innovation than using existing technology to enable our target users, HCPs, to provide better opioid-risk-informed pain management.

In this study, we tested the use of DS-RPM by HCPs for functionality, usability, and acceptability using three headache vignettes. Participant evaluations of usability and acceptability of DS-RPM were high. The qualitative data from early beta testing guided the functionality and user interface, especially structured data entry that we used in late beta testing. Participants stated that they would use DS-RPM in both education and clinical practice and recommend it to others. Below, we address research issues related to methodology, using ICHD-3 as a diagnostic knowledge source, and quantitative user evaluation, alluded to earlier.

### Methodology: Ranking Diagnoses and Handling Multiple Diagnoses

Varied computational approaches have been used to handle unclear diagnoses or multiple simultaneous diagnoses. Early decision support systems such as Iliad system [59] used Bayesian approaches with prior probabilities, grounded on baseline prevalence of candidate diseases both by themselves and conditional to the presence of specific findings to rank candidate diagnoses. However, even if computable from local EHR data, incidence varies by geographic locale, and so ranking is only a crude guide. In other words, a candidate diagnosis’s probability must drop below a very small threshold value to be ruled out.

The creators of Internist-1 [60] and its successor, Quick Medical Reference (QMR) [61], the most comprehensive CDS tools in existence, used two ordinal measures for prioritization:

1. *Evoking strength*, progressing from a symptom/finding to a disease, corresponds approximately to precision*.* Ranking includes 0 = nonspecific finding; 1 = presence of finding minimally suggests this disease; … 5 = presence of finding always suggests this disease*.*
2. *Frequency value*, progressing from a disease to a finding, corresponds to sensitivity. Ranking includes 1 = if this disease is present, this finding is rarely seen; … 6 = if disease is present, this finding is always seen.

QMR could also segregate findings into clusters. Each cluster was explained by a separate disease.

Our task is somewhat different and simpler in primary care, especially in urgent care, rural locales, and satellite clinics. Here, available EHR information on investigations is limited to previously seen patients and may not necessarily apply to present complaints. So, workup is less comprehensive than in secondary/tertiary-care centers. In such settings, DS-RPM may prove most valuable because patients often receive supportive therapy before referral or are often emergently sent to advanced care.

Also, ranking diagnoses probabilistically may be premature when there is limited information available, especially in complex cases. Rather than using probabilities, DS-RPM presents differential diagnoses and suggests workup related to differentiating clinical findings and investigations. Differential diagnosis, employed long before electronic computing, is a fundamental step in clinical reasoning and provides the framework to let clinicians think methodically [62].

### ICHD-3 Limitations

We found ICHD-3 useful initially, but later discovered the following limitations related to clinical practice. While it is possibly unfair to criticize a source created mainly for taxonomic purposes, clinicians and researchers addressing headache and facial pain may benefit from knowing these issues.

1. ICHD-3 does not address differential diagnosis, intermediate diagnoses, or treatment.
2. Multiple simultaneous headache disorders are not addressed. Thus, an ICHD-3 diagnostic criterion for tension-type headache (TTH), the most common headache disorder, is *absence* of nausea/vomiting. However, migraine, where nausea/vomiting is prominent, often co-occurs with TTH. This well-known clinical combination (“Mixed Tension-Migraine”) [63] is surprisingly omitted from ICHD-3. Therefore, this ICHD-3 criterion for TTH diagnosis has limited practical utility.
3. Some conditions, such as TTH, are sub-classified at excessively fine granularities that are marginally important to therapeutics.

### Usability/Acceptability Evaluation

We decided to adapt and abbreviate the 10-question System Usability Scale (SUS), while using the same Likert scale. The reasons follow:

- *SUS has considerable redundancy*: Several questions elicit the same semantic information. Thus, Question 5, “I found the various functions in this system were well integrated” is the reverse of Question 6, “I thought there was too much inconsistency in this system”.
- In software contexts, “inconsistency” means having to perform conceptually similar or identical tasks using different keyboard/mouse actions in different parts of the system. This happens when separate developer teams working on a large system fail to coordinate their user-interface efforts. Conversely, in “well-integrated” software, users access diverse functionality using a common, memorable, user-interface metaphor.
- *SUS redundancy leads to highly correlated responses*, suggesting opportunities for abbreviation [64]. Principal components analysis of SUS [65, 66] found two components, *“learning*” and “*usability.*” Referring to Table 2, we capture learnability and usability with questions B2/B3, respectively.
- Our participants have sufficient clinical expertise to critique DS-RPM. Therefore, via questions B1/B4, we needed to capture an additional usability dimension, *understandability*. Participants will not accept “because-I-say-so”-type explanations.
- SUS lacks a counterpart to “I would recommend (DS-RPM) to others.”

## Limitations and Future Directions

While our results are encouraging, there are limitations of this work. First, the number of testers was small (early beta, *n* = 3; late beta, *n* = 21). However, three dedicated testers are appropriate for early beta with the objective to catch as many obvious bugs as possible. Also, the Food and Drug Administration (FDA) recommends at least 15 participants per user population to identify 90–97% of usability issues [67, 68]. Second, while DS-RPM handles numerous diagnoses, participants tested only three diagnoses. More diagnoses would be evaluated during real-time use with actual patients. Third, using a five-point Likert scale to evaluate usability and acceptability has limitations. A Likert scale represents a subjective response to a question on an ordinal scale, with anchoring phrases to define each point on the scale. To summarize our findings, we treated these data as interval data rather than ordinal data [69, 70]. Means and standard deviations were used because they provided interpretable summary of responses.

Based on the feasibility study’s results, we may take DS-RPM in several future directions:

- *Extending DS-RPM’s knowledge content* to address thoraco-abdominal pain. While considerable effort would be needed, DS-RPM’s diagnosis-agnostic architecture allows us to add new clinical variables, diagnoses, differentials, and treatments. However, the interactions with existing content must be tested thoroughly, e.g., for diagnoses like abdominal migraine and multi-system disorders.
- *Upsizing* DS-RPM to a multi-tier architecture with a high-end database and a Web front-end.
- *Inter-operating* more closely with the EHR, using FHIR, which is Fast Healthcare Interoperability Resources technology [71]. This goal is distant because it involves several moving parts, e.g., institutional buy-in. As a first step, open-sourcing the code and thoroughly documenting DS-RPMs internals will allow others to replicate it for their needs.

## Conclusion

The decision support for responsible pain management (DS-RPM) was designed, created, tested, and revised to facilitate best practice in the management of patients with headache and facial pain. Testing the DS-RPM with vignettes showed strong functionality, high ratings on usability, and high ratings on acceptability from HCPs. Risk stratifying for opioid use disorder to develop a treatment plan for headache and facial pain is possible using vignettes. During testing, we considered issues related to handling unclear and multiple diagnoses, and the need to adapt usability/acceptability evaluation tools for clinical decision support. These test results indicate DS-RPM is ready for the next phase of testing with HCPs using DS-RPM in patient encounters.

## Data Availability

The datasets analyzed during the current study are available from the corresponding author on reasonable request.

## Abbreviations

DS-RPM: Decision Support for Responsible Pain Management
HCP: Health care providers
CaMEO: Chronic Migraine Epidemiology and Outcomes
OUD: Opioid use disorder
CDS: Clinical decision support
EHR: Electronic health record
ICHD-3: International Classification of Headache Disorders, 3rd Edition
ORT-OUD: Opioid Risk Tool for Opioid Use Disorder
ASQ: Ask Suicide Screening Questions
PHQ-9: Patient Health Questionnaire, 9 items
SQL: Structured Query Language
GUI: Graphical user interface
DEA: Drug Enforcement Agency
SUS: System Usability Scale
QMR: Quick Medical Reference
TTH: Tension-type headache
FHIR: Fast Healthcare Interoperability Resource
